# Bacteria Remediate the Effects of Food Additives on Intestinal Function in an *in vitro* Model of the Gastrointestinal Tract

**DOI:** 10.3389/fnut.2020.00131

**Published:** 2020-08-12

**Authors:** Mridu Malik, Sanjeena Subedi, Cláudia N. H. Marques, Gretchen J. Mahler

**Affiliations:** ^1^Department of Biomedical Engineering, Binghamton University, Binghamton, NY, United States; ^2^Binghamton Biofilm Research Center, Binghamton University, Binghamton, NY, United States; ^3^Department of Mathematical Sciences, Binghamton University, Binghamton, NY, United States; ^4^Department of Biological Sciences, Binghamton University, Binghamton, NY, United States

**Keywords:** Caco-2, HT29-MTX, *in vitro* digestion, microbiota, food additives, brush border enzyme, nanoparticles

## Abstract

As the site of nutrient absorption, the small intestine is continuously exposed to preservatives and additives present in consumed food. While the effects of diet on the lower gastrointestinal tract are widely studied, the effects of food additives on the small intestinal epithelium and microbiota are less clearly understood. The goal of this work was to develop and establish a physiologically relevant model of the upper gastrointestinal tract to study the complex interactions between food additives, individual bacterial species, and intestinal function. To achieve this, an *in vitro* model incorporating simulated digestion, human intestinal epithelial cells, and the commensal, Gram-positive *Lactobacillus rhamnosus*, or the opportunistic, Gram-negative *Escherichia coli* was developed. This model was used to assess intestinal permeability and alkaline phosphatase activity following exposure to high glucose (HG), salt, emulsifier (TWEEN 20), food (milk chocolate candies) or chemical grade titanium dioxide nanoparticles (TiO_2_-NP), and food (whole wheat bread) or chemical grade gluten. It was found that HG increased intestinal permeability, the presence of bacteria remediated the negative effects of HG on intestinal permeability, and a decrease in permeability and IAP activity was observed with increasing concentration of TWEEN 20 both in the presence and absence of bacteria. While *L. rhamnosus* influenced the activity of intestinal alkaline phosphatase and tight junction protein distribution, *E. coli* produced indole to reinstate intestinal permeability. The source of TiO_2_ and gluten led to altered impacts on permeability and IAP activity. The growth of *E. coli* and *L. rhamnosus* was found to depend on the type of food additive used. Overall, the presence of bacteria in the *in vitro* model influenced the effects of food additives on intestinal function, suggesting a complex association between diet and upper GI microbiota. This model provides a method to study small intestinal function and host-microbe interactions *in vitro* in both healthy and diseased conditions.

## Introduction

The gastrointestinal (GI) tract, the most heavily colonized organ, contains more than 70% of all microorganisms inhabiting the human body (1). Most published research focuses on the significance and involvement of the large intestinal microbiome in overall gut and/or host health. In the lower GI tract, microorganisms are known to be involved in biosynthesis of essential amino acids and vitamins and production of byproducts from undigested metabolite such as short chain fatty acids that can strengthen the mucosal barrier (2). The human small intestine (SI) provides the transition between the stomach and the large intestine. It is the site of absorption for most nutrients and minerals (3). The SI is colonized by an abundant variety of beneficial and potentially opportunistic microorganisms that reside near the host cells. The microbiota present in the intestinal lumen are separated from the outside environment by the semi-permeable intestinal barrier. While the upper small (proximal) intestine contains 10^1^-10^3^ colony forming units (CFU) per mL, the number of viable organisms increases to 10^4^-10^7^ CFU/mL in the distal portion of the SI (1). The acid, pancreatin, and bile secretions from the digestion process contribute to the limited bacterial colonization in the proximal SI (4). The SI is predominantly rich in Gram-positive bacterial species such as *Lactobacillus* (1, 4). Some pathogenic strains of *E. coli*, a Gram-negative bacterium, can also adhere and be present in the small intestine (5). The SI barrier selectively allows the transport of nutrients while restricting the passage of pathogens into the blood stream through production of mucins and antimicrobial proteins.

Although GI disorders are complex, the increased use and corresponding consumption of food additives and preservatives has corresponded to a proportional increase in occurrence of GI disorders, such as irritable bowel syndrome (IBS), and inflammatory bowel disease (IBD) (6). The intestine of certain IBS patient groups display low-grade inflammation and downregulation and redistribution of Zonula occludens-1 (ZO-1) tight junction proteins (7). A dysfunctional epithelial barrier, as observed in these disorders, leads to an altered permeability, and intestinal alkaline phosphatase (IAP) activity (7, 8). IAP is a gut mucosal defense factor, is located on the apical membrane of the intestinal barrier and is involved in regulation of the intestinal epithelial barrier through interactions with the microbiome. Under normal conditions, IAP protects the host during inflammation by de-phosphorylation of lipopolysaccharides (LPS), a component of the outer membrane of Gram-negative bacteria (9).

In this study, a co-culture of Caco-2 and HT29-MTX was used as an *in vitro* intestinal model. Caco-2 cells are derived from colonic epithelial adenocarcinoma cells with the ability to differentiate into enterocyte-like epithelial barrier. These cells polarize and express microvilli, glucose transporter proteins (GLUT1, −3, −5) (10), and tight junctions (TJ), and produce brush border membrane (BBM) enzymes such as IAP (11). The HT29-MTX cells are derived from human colonic adenocarcinoma cells adapted to methotrexate (MTX). These cells differentiate into goblet-like cells, and produce a protective mucus layer on the enterocyte monolayer (12). The co-culture model was exposed to commonly used food additives such as digested TiO_2_ nanoparticles (NP), and gluten and non-digested high glucose (HG), salt, and emulsifiers. In addition to tests using pristine TiO_2_ NP and laboratory grade gluten, these additives were also tested from commercial food sources. The effects on the integrity of the epithelial barrier were assessed in combination with tumor necrosis factor (TNFα) (to model an inflammatory environment) and the commensal Gram-positive bacteria (*Lactobacillus rhamnosus*) or the opportunistic Gram-negative bacteria (*Escherichia coli*). The inclusion of these two types of bacteria with the mammalian model forms a basis for understanding the interaction between bacterial and mammalian cells in the small intestinal environment. This study provides insight into host-bacterium-diet interactions and food additive effects on small intestinal function in an *in vitro* model. Compared to lower GI microbiome characterization, which is based on stool samples, it is very challenging to obtain samples from the upper GI. The small number of studies that have attempted to characterize the SI microbial composition focus on the diseased state of the SI, making it difficult to estimate the normal conditions. There is a current need to understand the complex interaction between small intestinal function, single bacterial species, and food ingestion and this model provides a proof of concept method to conduct this work. This study also presents a direct comparison between the effects of the laboratory-grade, chemical form of additives and the food grade additives within a food matrix as consumed by the general population.

## Materials and Methods

### Intestinal Cell Co-culture

Caco-2 cells were purchased from American Type Culture Collection at passage 17 and used for experimentation at passage 45–55. The HT29-MTX intestinal epithelial cell line was kindly provided by Dr. Thécla Lesuffleur of INSERM U560 in Lille, France, at passage 11 and used at passage 40–50 (13). The two cell types were grown, and maintained, in Dulbecco's Modified Eagle Medium (DMEM, Thermo Fisher Scientific) containing 4.5 g/L glucose and 10% (v/v) heat inactivated fetal bovine serum (HI-FBS, ThermoFisher Scientific) and maintained in a 5% CO_2_ incubator at 37°C. The culture medium was changed every other day. Upon reaching 80% confluency, the cells were passaged and seeded onto polycarbonate, 0.4 μm pore size, 0.33 cm^2^ membrane, Transwell inserts for permeability studies, or onto 24-well culture plates for IAP activity assays, or onto sterilized glass cover slips in a six-well plate for immunocytochemistry. The wells were coated with rat tail Type I collagen (BD Biosciences) at a concentration of 8 μg/cm^2^ for 1 h at room temperature. To implement the intestinal monolayer, the cells were stained with trypan blue, counted, and seeded at a density of 10^5^ cells/cm^2^ and a ratio of 3:1 (Caco-2:HT29-MTX). The cells were grown for 14 days to allow for the formation of a mucus layer before experiments were conducted. For the high glucose study, both cell lines were adapted to low glucose medium by culturing the cells for 5 passages in glucose-free DMEM (ThermoFisher Scientific) supplemented with 5 mM sterilized glucose (to represent the physiological glucose level) and 10% (v/v) HI-FBS. Following adaptation to low glucose conditions, cells were seeded for experiments as described above.

Transepithelial resistance (TEER) was measured prior to all permeability assays using an EVOM2 with the Endohm-6 chamber from World Precision Instruments. The Endohm chamber was soaked in 70% ethanol for 15 min, then 2 mL of sterile 100 mM KCl solution was added to the chamber, and it was connected to the EVOM2. A sterilized Calicell (WPI) with 200 μL KCl was then used to calibrate the chamber. Finally, the Endohm chamber was rinsed with sterile 18 MΩ water and equilibrated with 2 mL serum-free DMEM for 15 min before TEER was measured for each Transwell membrane. Membranes with a TEER of 250–300 Ω^*^cm^2^ were used for experiments.

### Bacterial Cell Cultures

*Escherichia coli* (ATCC 11775) and *Lactobacillus rhamnosus GG* (ATCC 53103), both originally isolated from humans, were purchased from American Type Culture Collection. A tri-culture model was constructed by introducing *E. coli* or *L. rhamnosus* [described by (14)] into the Caco-2/HT29-MTX co-culture model. Overnight cultures of *E. coli* were grown in nutrient broth (Difco^TM^) while *L. rhamnosus* were grown in MRS broth (Difco^TM^). Prior to inoculation, bacterial concentrations of the overnight cultures were estimated using OD_600_, using an established calibration curve. Bacteria were introduced into the wells or apical chamber of the Transwells, in high or low glucose DMEM, at a concentration of 10^3^ or 10^4^ CFU/mL. For combination studies with bacteria and food additives, the bacterial inoculum concentrations were prepared in the respective food grade or chemical grade samples. Establishment of the calibration curve was obtained by correlating the absorbance with viable counts (CFU/mL). Each bacterial culture was serially diluted, and to each dilution, measurements of OD_600_ and viable counts were performed. Viable counts were performed by drop plating each dilution of *E. coli* onto nutrient agar (Difco^TM^) and *L. rhamnosus* onto MRS Agar (Difco^TM^), which were then incubated for 24 to 48 h, at 37°C with 5% CO_2_.

### Food Grade Sample Preparation

Cell cultures were exposed to TiO_2_ NPs as previously described (15), and to gluten within a food matrix. Weir et al. previously reported M&M chocolate candies to be one of the top 10 products with the highest TiO_2_ content (16). Nature's Harvest 100% whole wheat bread was used as a gluten source due to its high protein content, while Ener-g's gluten free bread served as the gluten control. The average gluten consumption in a “western diet” is 5–15 g/day and gluten content as low as 50 mg is also harmful to patients with celiac disease (CD) (17). In this study the gluten concentration was used at 50 mg/well and a concentration of 250 mg/mL. These values were based on the nutrition label on the bread where 1 slice of whole wheat bread weighing 29 g consists of 3.5 g of protein.

The M&Ms and bread were first freeze dried for 72 h, to remove all the water content from the food products. The freeze-dried samples were then separately crushed in a mortar pestle, weighed, and then digested *in vitro* using previously described methods (18). Briefly, the *in vitro* digestion protocol entailed subjecting the weighed samples to “gastric” digestion by adding 20 mL of 140 mM NaCl + 5 ml KCl (prepared at pH 2) solution to each sample and readjusting the pH 2 with 1 M HCl. Next, 1 mL porcine pepsin solution (25 mg/mL, 800–2,500 U/mg protein, Sigma-Aldrich) in 0.1 M HCl, was added to each sample, and the samples were rocked at 55 oscillations/min for 1 h at 37°C with 5% CO_2_. The rocking action mimics the digestion movement in the human stomach. After the 1 h incubation, as part of the “intestinal” digestion, the pH of the samples was raised to 5.5–6.0 by adding 0.1 M or 1 M NaHCO_3_, and 5.5 mL of porcine pancreatin-bile solution consisting of 2 mg/mL pancreatin (a mixture of trypsin, amylase, lipase, ribonuclease, and protease activities, Sigma P3292) and 11 mg/ml bile extract (a mixture of glycine and taurine conjugates of hyodeoxycholic and other bile acids, Sigma B8631) was added. The pH was then adjusted to 7.0 and the volume of each tube was brought to 30 mL by weight with 140 mM NaCl + 5 mM KCl solution, prepared at pH 6.7. The samples were then referred to as digests and used for exposure studies.

### Chemical Grade Sample Preparation

Solutions (v/v) of 0.01, 0.1, and 1% NaCl (salt, Sigma) and TWEEN 20 (Sigma) were freshly prepared in DMEM and 0.2 μm syringe filter-sterilized prior to the experiments. The sample wells were exposed to 500 μL of 0.01, 0.1, and 1% salt or TWEEN 20 solution for IAP study. The permeability study was conducted in Transwells by adding 200 μL of 0.01 and 1% salt or TWEEN 20 solution in the apical chamber and 600 μL of DMEM in the basolateral chamber. DMEM with 10% HIFBS was used as a control for each of these conditions. For high glucose exposure study, the Transwell plates previously seeded with low glucose cells were first washed with 1 × PBS. The sample wells were exposed to 200 μL of HG (25 mM glucose DMEM) solution in the apical chamber while the basolateral chamber was filled with 600 μL of 5 mM glucose DMEM. For the control wells, 200 μL of control solution (5 mM glucose + 20 mM mannitol) was added to the apical chamber and 600 μL of 5 mM glucose DMEM was added to the basolateral chamber. To prepare chemical grade TiO_2_ and gluten samples, the experimental concentrations were kept the same as that for food grade sample preparations. Gluten and previously characterized pristine, 30 nm anatase TiO_2_-NPs (19) (US Research Nanomaterials, Inc.) was weighed, and dissolved in DMEM. The prepared solutions were then digested *in vitro* according to the protocol described in the previous section. DMEM, digested *in vitro*, served as a control [referred to as TiO_2_ control (ch) or gluten control (ch)]. In addition, to compare the effect of the digestion protocol on experiments, non-digested DMEM was used as an overall control.

### Inflammation

To mimic inflammatory conditions, TNFα, a pro-inflammatory stress mediator, was prepared in 1 × PBS. The cell culture model was exposed to 10 ng/mL of TNFα for 24 h prior to exposure to food digests.

### Permeability Assay

The changes in permeability across the *in vitro* monolayer were detected using Lucifer Yellow (LY) (Sigma), a fluorescent paracellular permeability marker. A stock of 1 mg/mL was prepared in 18 MΩ water and stored at 4°C until needed. The permeability study was conducted for 4 h. For the experiments, 100 μL of 50 μM LY were added to the apical chamber of Transwells along with the different samples. A sample of 100 μL was taken from the basolateral chamber every 15 min for the first hour and every 30 min for the subsequent 3 h. Each sampling was coupled with the addition of 100 μL of fresh DMEM (or low glucose DMEM for HG study) to the wells. The samples were collected in a 96-well opaque black bottom plate (Corning). At the end of the exposure experiment, the 96-well plate was read using a Synergy 2 plate reader, controlled by Biotek's Gen5™ Reader Control and Data Analysis Software. The resulting fluorescence values were converted to amount of LY (μg) using a LY standard curve.

### Bacterial Viability Assay

A 100 μL sample of bacteria in culture medium or food digest was taken from the apical Transwell chamber post-permeability study. The samples were serially diluted in 0.9% sterile saline solution and 10 μL of each dilution were plated in triplicate on agar plates and quantified as described in 2.2.

### Indole Quantification

To estimate the amount of indole produced by *E. coli* under each condition, Kovac's Indole test was used. Post-exposure, 100 μL sample was collected from the apical chamber and added into a 96-well clear bottom plate after which, 150 μL of Kovac's reagent (Sigma) was added to each well. The plate was incubated for 30 min at room temperature and then read at 530 nm using Synergy 2 plate reader, controlled by Biotek's Gen5™ Reader Control and Data Analysis Software. An indole standard curve was prepared to estimate the amount of indole produced under different conditions.

### Alkaline Phosphatase Assay

IAP activity was estimated through IAP and Bradford assays performed on the monolayers grown in 24-well plates. The wells were exposed to sample or control solutions for 4 h. At the end of the exposure period, the wells were washed with 1 × PBS and 200 μL of 18 MΩ water was added to each well. The plate was sonicated at 4°C for 15 min before transferring the content of each well into different 0.5 ml microcentrifuge tubes. For the IAP assays, 85 μL of p-Nitrophenyl Phosphate (pNPP, Sigma) solution and 25 μL of sonicated sample or IAP standard (prepared using *p*-nitrophenol and pNPP) was added to each well of a 96-well clear bottom opaque side plate and allowed to incubate at 37°C for 1 h. For the Bradford assay, 250 μL of Bradford reagent (Sigma) and 5 μL of sonicated sample or Bradford standard (prepared using Bovine Serum Albumin (BSA) and 18 MΩ water) was added to the wells. The Bradford assay plate was incubated at room temperature for 30 min. The IAP and Bradford assay plates were read at 405 and 595 nm, respectively, using Synergy 2 plate reader, controlled by Biotek's Gen5™ Reader Control and Data Analysis Software. Using the data, mg IAP/mg total cell protein was calculated and plotted.

### Immunocytochemistry

The Caco-2 and HT29-MTX cell monolayer was formed on sterile cover slips in 6-well plates. The co-culture model was exposed to HG or glucose control (5 mM glucose + 20 mM mannitol), and 0.01, 0.1, 1% salt or control (DMEM) for 4 h. The additives were used in combination with 10^3^ CFU/mL of *E. coli* or *L. rhamnosus*. Post exposure, cells were fixed with 4% paraformaldehyde (PFA) for 15 min, followed by incubation with 0.2% Triton X-100 (prepared in PBS) for 10 min. Cells were then subjected to a blocking agent (1% BSA in PBS) for 1 h at 4°C. The wells were then washed with PBS and incubated with mouse anti-occludin (Invitrogen, # 33-1500, 1:100 in PBS), and rabbit anti-zonulin 1 primary antibodies (Abcam, ab59720, 1:100 in PBS) for 1 h at 4°C. The cells were washed again and incubated with Alexa Fluor 488 goat anti-mouse (Thermo Fisher Scientific # A32723, 1:100 in PBS) and Alexa Fluor 568 goat anti-rabbit (Thermo Fisher Scientific # A11011, 1:100 in PBS) secondary antibodies for 2 h in the dark at room temperature. The DNA of cells was stained using DRAQ5 (Thermo Fisher Scientific, 1:1000 in PBS). Finally, the cells were washed with 18 MΩ water and mounted onto glass slides with ProLong gold mounting medium (Thermo Fisher Scientific) and left in the dark overnight. Cells were imaged with a Zeiss LSM 880.

### Statistical Analysis

The datasets were transformed using Box-Cox transformations, as appropriate. The transformed datasets were tested for normality using Shapiro-Wilk test. A linear regression model was fitted to every pair of conditions in the Lucifer yellow permeability data. A hypothesis test was conducted to test whether the linear regression model for the pairs of conditions had the same slopes and intercepts, followed by false discovery rate (FDR) correction. FDR correction was done using Benjamini and Hochberg procedure (20) implemented in the R (21) package multtest (22). The IAP assay, and bacterial viability results were compared using two-way ANOVA using GraphPad Prism 8.3.1, followed by FDR correction to account for multiple hypothesis tests. Differences were considered significant at FDR-adjusted *p* < 0.05. Immunohistochemistry images were analyzed using ImageJ (23). The level of tight junction protein fluorescence was determined by intensity and surface area profiling. The background intensity was removed by thresholding. ZO-1 and occludin distribution were compared based on the surface area covered by each protein per number of cells in the frame. The results were analyzed using a two-way ANOVA followed by FDR correction.

## Results

### Changes in Permeability and/or Intestinal Alkaline Phosphatase With Glucose, Salt, and TWEEN 20

**Figure 1:**
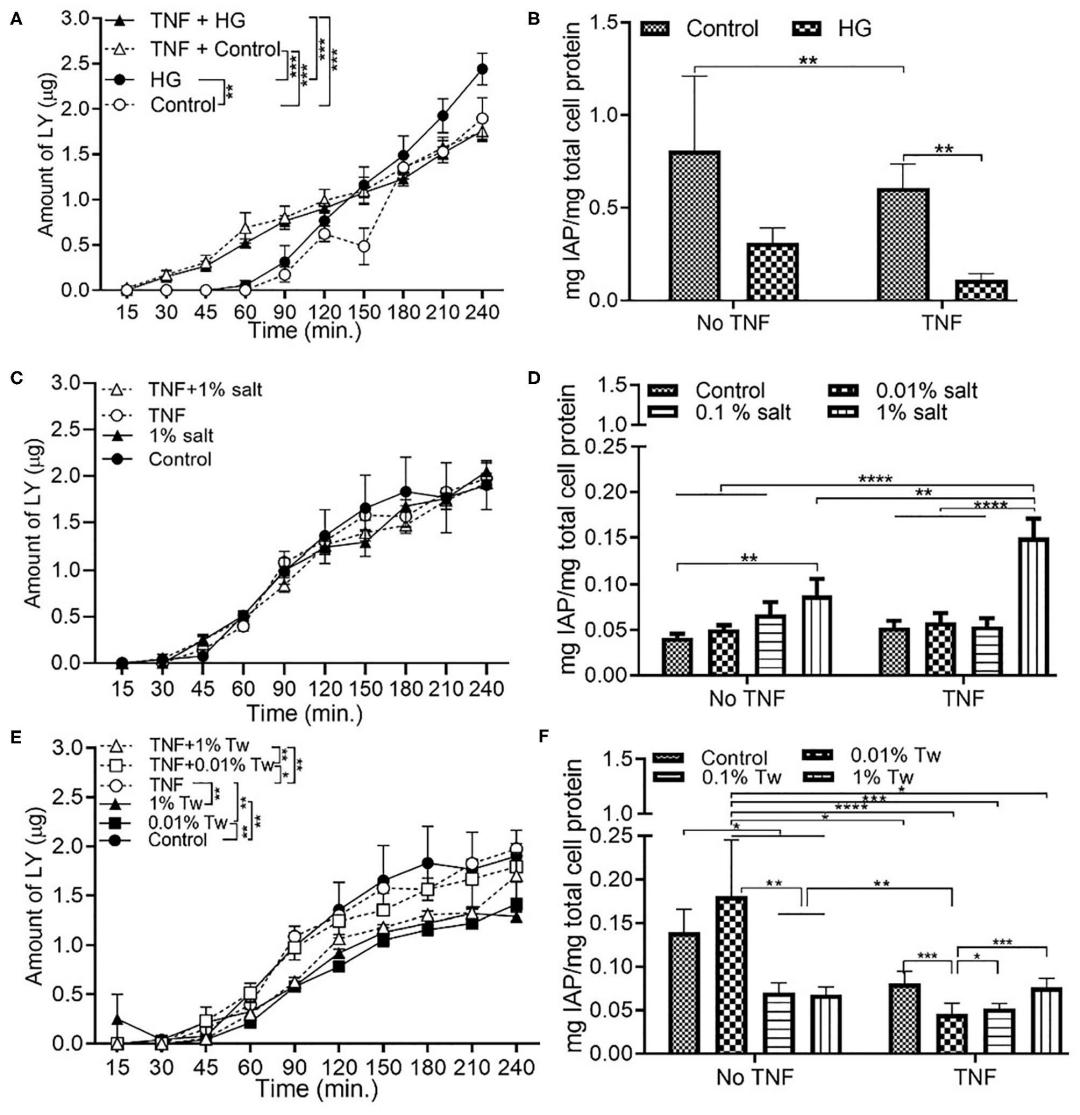
Changes in cell permeability and/or intestinal alkaline phosphatase with glucose, salt, and TWEEN 20. The Caco-2/HT29-MTX intestinal model was exposed to 10 ng/mL of TNF-α (TNF) for 24 h and different food additives for 4 h. Control (5 mM glucose + 20 mM mannitol) and high glucose (25 mM DMEM, HG) **(A)** Permeability **(B)** IAP activity. Control (DMEM), 0.01, 0.1, and 1% salt **(C)** Permeability **(D)** IAP activity. Control (DMEM), 0.01, 0.01, and 1% TWEEN 20 (Tw) **(E)** Permeability **(F)** IAP activity. Error bars represent SEM. FDR-adjusted *p*-values: *P < 0.05; **P < 0.01; ***P < 0.001; ****P < 0.0001 using linear regression with FDR correction for permeability assay and two-way ANOVA with FDR correction for IAP assay. The data was collected from two experimental replicates with four (permeability assay) and 12 (IAP assay) biological replicates each. Tw, TWEEN 20.

Exposure of Caco-2/HT29-MTX monolayer to HG resulted in a significant increase in permeability and decrease in IAP after the 4 h exposure, when compared to the control conditions. Addition of the inflammation mediator, TNFα, led to reduced permeability, and IAP activity in the HG conditions (Figures 1A,B). Compared to control (DMEM), 1% salt as an additive did not change the permeability with or without TNFα at the end of the exposure time (Figure 1C). A lower salt concentration of 0.01% did not show a significant difference compared to control (Figure S1A). However, an increase in IAP activity with increased salt concentration was observed, both in presence and absence of TNFα (Figure 1D).

On the other hand, permeability (Figure 1E), and IAP activity (Figure 1F) decreased with increasing concentration of TWEEN 20 compared to the controls (DMEM). An exception to this was the presence of 0.01% TWEEN 20 where the highest IAP activity was observed in absence of TNFα. TNFα led to reduced permeability in HG exposures, and an increase in permeability when in combination with TWEEN 20.

### Food Grade Gluten Exposure Leads to Higher Permeability Than Food Grade TiO_2_

**Figure 2:**
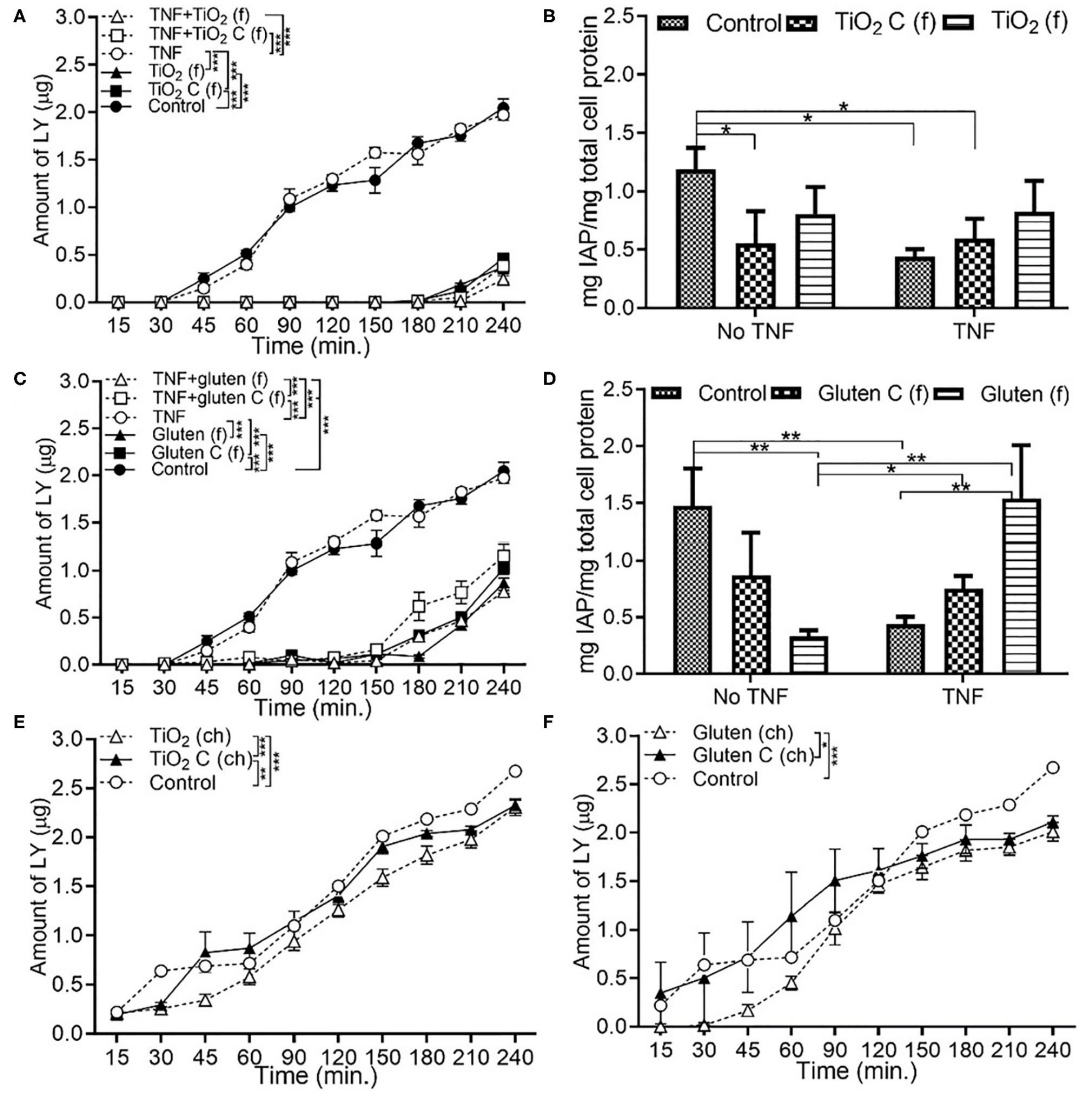
Food grade gluten exposure leads to higher monolayer permeability than food grade TiO_2_. The Caco-2/HT29-MTX intestinal model was exposed to 10 ng/mL of TNFα (TNF) for 24 h and medium (DMEM subjected to *in vitro* digestion, serves as overall control) or food grade (f) or chemical grade (ch) additives for 4 h. TiO_2_ (f) or TiO_2_ C (f) (M&M candies without outer TiO_2_ shell) **(A)** Permeability **(B)** IAP activity. Gluten (f) and gluten control (f) (gluten free bread), **(C)** Permeability **(D)** IAP activity. TiO_2_ (ch) and TiO_2_ control (ch) **(E)** Permeability. Gluten (ch) and gluten control (ch) **(F)** Permeability. Error bars represent SEM. FDR-adjusted *p*-values: *P < 0.05; **P <0.01; ***P <0.001 using linear regression with FDR correction for permeability assay and two-way ANOVA with FDR correction for IAP assay. The data was collected from two experimental replicates with 4 (permeability assay) and 12 (IAP assay) biological replicates each. C, control.

In the presence of food grade additives, there was a significant decrease in permeability with TiO_2_ and TiO_2_ control, both with and without TNFα compared to DMEM controls (Figure 2A). This was associated with a decrease in IAP activity with pre-exposure to TNFα (Figure 2B). In the food grade gluten, there was a significant decrease in permeability in the presence of gluten compared to DMEM controls (Figure 2C). Exposure to food grade gluten showed a higher permeability compared to food grade TiO_2_, suggesting a more negative impact on the intestinal barrier integrity. Exposure to food grade gluten lowered the IAP activity compared to DMEM controls. The IAP activity reverted to control levels upon exposure to TNFα + food grade gluten (Figure 2D). One main aspect of this study was to determine whether a single type of food additive can lead to different effects on intestinal function when in its chemical or food grade form. For this, TiO_2_ and gluten were also used in their chemical form. Chemical grade TiO_2_ (Figure 2E) and gluten (Figure 2F) led to a decrease in permeability compared to controls. Overall, the permeability was significantly lower in chemical grade TiO_2_ and gluten exposure conditions than food grade conditions.

### *E. coli* Decrease Permeability and IAP Activity in HG and TWEEN 20 Condition

**Figure 3:**
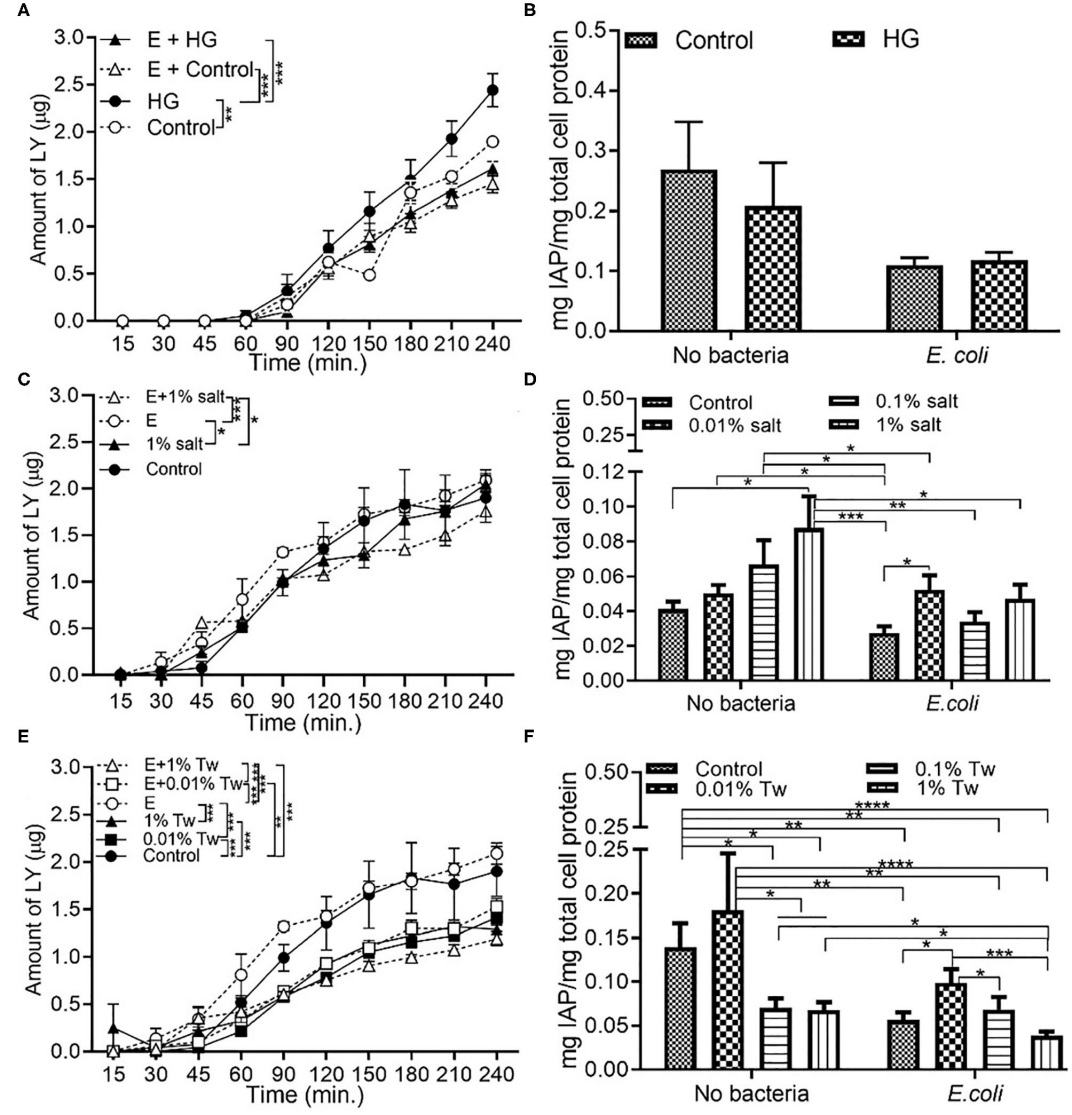
*E. coli* decreases monolayer permeability and IAP activity in HG and TWEEN 20 condition. The Caco-2/HT29-MTX intestinal model was exposed to different additives and 10^3^ CFU/mL *E. coli* for 4 h. Control (5 mM glucose + 20 mM mannitol) and high glucose (25 mM DMEM, HG) **(A)** Permeability **(B)** IAP activity. Control (DMEM), 0.01, 0.1, and 1% salt **(C)** Permeability **(D)** IAP activity. Control (DMEM), 0.01, 0.01, and 1% TWEEN 20 (Tw) **(E)** Permeability **(F)** IAP activity. Error bars represent SEM. FDR-adjusted *p*-values: *P < 0.05; **P < 0.01; ***P < 0.001; ****P < 0.0001 using linear regression with FDR correction for permeability assay and two-way ANOVA with FDR correction for IAP assay. The data was collected from two experimental replicates with four (permeability assay) and 12 (IAP assay) biological replicates each. Tw, TWEEN 20; E, *E. coli*.

Exposure to *E. coli*, the opportunistic bacteria used in this study, led to a significantly decreased of the permeability across the monolayer in high glucose and control conditions compared to the absence of bacteria (Figure 3A). While a decrease in IAP activity was also present in *E. coli* + HG conditions, there was no statistical significance (Figure 3B). Higher initial concentrations of *E. coli* (10^4^ CFU/mL) also resulted in a significant decrease in permeability under HG conditions (Figure S2A). The reduction in permeability was higher with 10^3^ CFU/mL bacteria than 10^4^ CFU/mL, contributing to the selection of 10^3^ CFU/mL as the bacterial inoculum concentration used in this study. Compared to the absence of bacteria, a decrease in permeability was observed upon exposure to *E. coli* in combination with 1% salt (Figure 3C), but not upon exposure to *E. coli* and 0.01% salt (Figure S1B). For IAP assays, the presence of *E. coli* reduced the IAP activity compared to the absence of bacteria (Figure 3D). Similar to section Food Grade Gluten Exposure Leads to Higher Permeability Than Food Grade TiO2, a dose dependent decrease in permeability (Figure 3E) and IAP activity (Figure 3F) was observed in TWEEN 20 conditions with *E. coli*. An exception to this was an increased IAP activity upon exposure to *E. coli* with 0.01% TWEEN 20, similar to the trend observed in the absence of bacteria.

### Chemical Grade Additives Induce Higher Permeability Than Food Grade Additives

**Figure 4:**
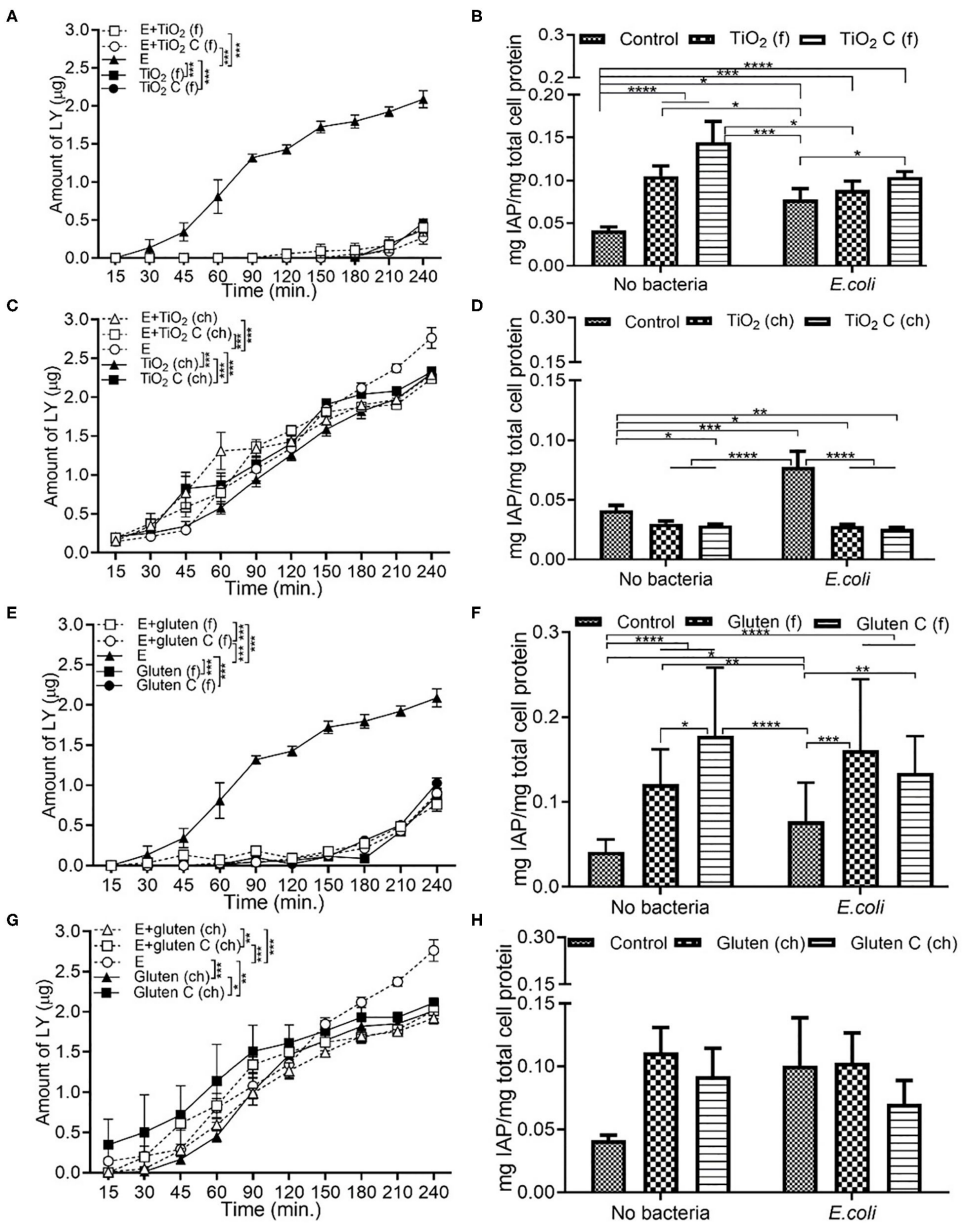
Chemical grade additives induce higher monolayer permeability than food grade additives. The Caco-2/HT29-MTX intestinal model was exposed to media (DMEM subjected to *in vitro* digestion, serves as overall control) or food grade (f) or chemical grade (ch) additives and 10^3^ CFU/mL *E. coli* for 4 h. TiO_2_ (f) or TiO_2_ control (f) **(A)** Permeability **(B)** IAP activity. TiO_2_ (ch) and TiO_2_ control (ch) **(C)** Permeability **(D)** IAP activity. Gluten (f) and gluten control (f) **(E)** Permeability **(F)** IAP activity. Gluten (ch) and gluten control (ch). **(G)** Permeability **(H)** IAP activity. Error bars represent SEM. FDR-adjusted *p*-values: *P < 0.05; **P < 0.01; ***P < 0.001; ****P < 0.0001 using linear regression with FDR correction for permeability assay and two-way ANOVA with FDR correction for IAP assay. The data was collected from two experimental replicates with four (permeability assay) and 12 (IAP assay) biological replicates each. C, control; E, *E. coli*.

Exposure to *E. coli* showed the highest permeability compared to combination with food additives. Compared to only *E. coli*, a combination of *E. coli* and food grade TiO_2_ decreased the permeability significantly (Figure 4A). In the case of IAP activity, both in the presence and absence of *E. coli*, a similar trend was observed. Food grade TiO_2_ and TiO_2_ control led to an increase in IAP activity, but with an overall reduced IAP level in presence of *E. coli* (Figure 4B). In terms of chemical grade TiO_2_, while *E. coli* still showed the highest permeability, the decrease in permeability with *E. coli* and chemical grade TiO_2_ was not as significant as in case of food grade TiO_2_ (Figure 4C). Compared to DMEM controls, IAP activity decreased upon exposure to chemical grade TiO_2_ and TiO_2_ control, both in presence and absence of *E. coli* (Figure 4D). Food grade gluten showed a low permeability both with and without *E. coli* (Figure 4E). While both TiO_2_ and gluten followed similar trend in terms of permeability, IAP activity showed different patterns on the basis of the source of additives (food or chemical grade). Food grade gluten had a positive effect on IAP activity both in the absence and presence of *E. coli* (Figure 4F). Compared to the absence of bacteria, *E. coli* did not contribute to any change in permeability when in combination with chemical grade gluten (Figure 4G). Chemical grade gluten with *E. coli* showed the lowest permeability of the other conditions in Figure 4G. Compared to DMEM controls, IAP activity increased with chemical-grade gluten exposure albeit not being significant (Figure 4H).

### *L. rhamnosus* Decrease the Permeability With HG and TWEEN 20

**Figure 5:**
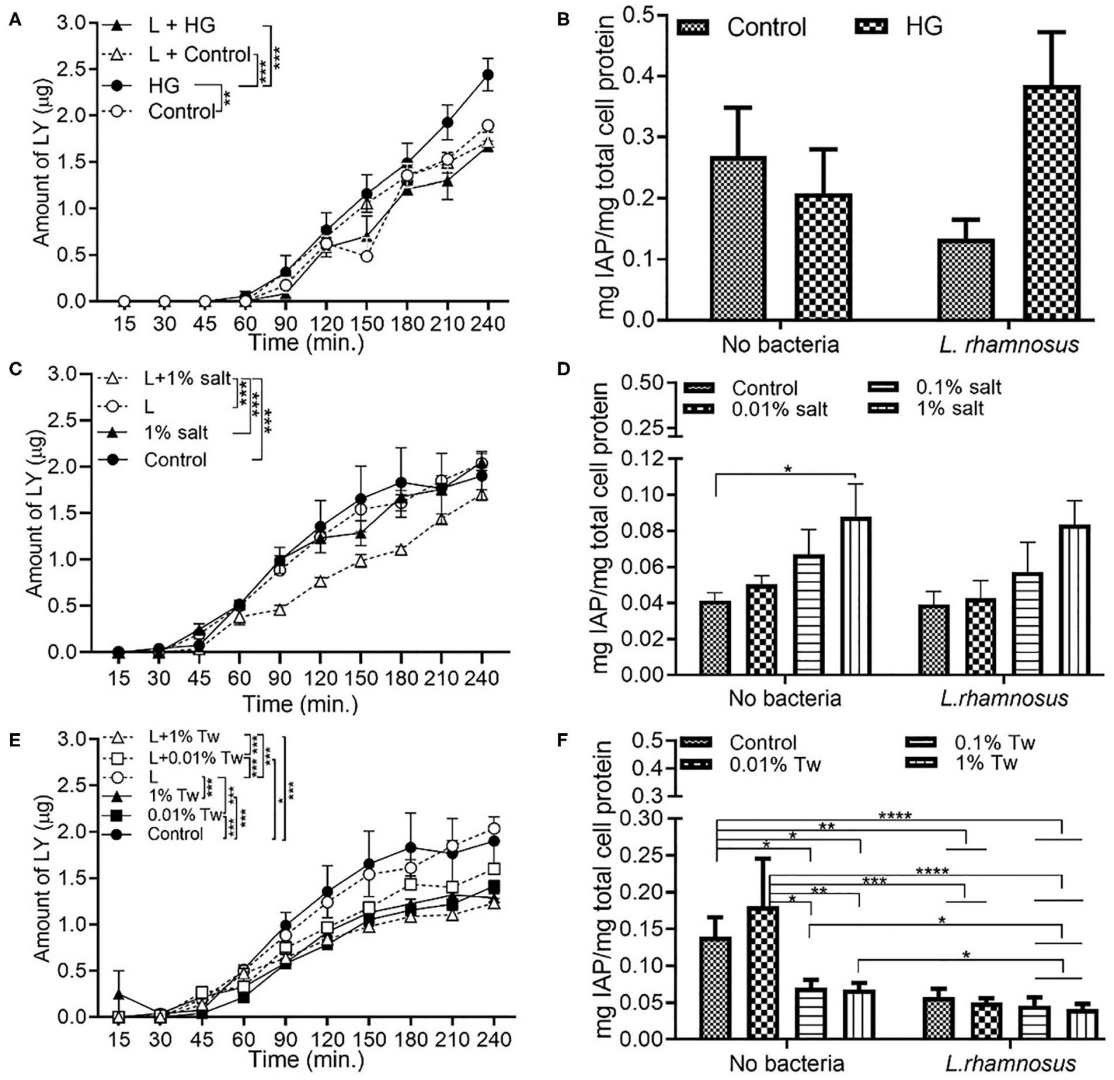
*L. rhamnosus* decreases the monolayer permeability with HG and TWEEN 20. The Caco-2/HT29-MTX intestinal model was exposed to different additives and 10^3^ CFU/mL *L. rhamnosus* for 4 h. Control (5 mM glucose + 20 mM mannitol) and high glucose (25 mM DMEM, HG) **(A)** Permeability **(B)** IAP activity. Control (DMEM), 0.01, 0.1, and 1% salt **(C)** Permeability **(D)** IAP activity. Control (DMEM), 0.01, 0.01, and 1% TWEEN 20 **(E)** Permeability **(F)** IAP activity. Error bars represent SEM. FDR-adjusted *p*-values: *P< 0.05; **P < 0.01; ***P < 0.001; ****P < 0.0001 using linear regression with FDR correction for permeability assay and two-way ANOVA with FDR correction for IAP assay. The data was collected from two experimental replicates with four (permeability assay) and 12 (IAP assay) biological replicates each. Tw, TWEEN 20; L, *L. rhamnosus*.

*L. rhamnosus*, modeling commensal bacteria, had the same effect on permeability when in combination with HG as the one observed for *E. coli*. The presence of *L. rhamnosus* led to a decrease in permeability when with HG (Figure 5A). Compared to the absence of bacteria, an increase in IAP was observed in *L. rhamnosus* and HG conditions, but without any statistical significance (Figure 5B). The introduction of *L. rhamnosus* into the *in vitro* model led to the highest permeability, which was reduced in combination with 1% salt (Figure 5C) and 0.01% salt (Figure S1C). IAP activity with *L. rhamnosus* and salt showed similar trend to the one observed in the absence of bacteria, albeit with no statistical difference (Figure 5D). *L. rhamnosus* led to a decrease in permeability (Figure 5E) and IAP activity (Figure 5F) when with TWEEN 20 compared to the absence of bacteria.

### Additive Source Affects the IAP Activity

**Figure 6:**
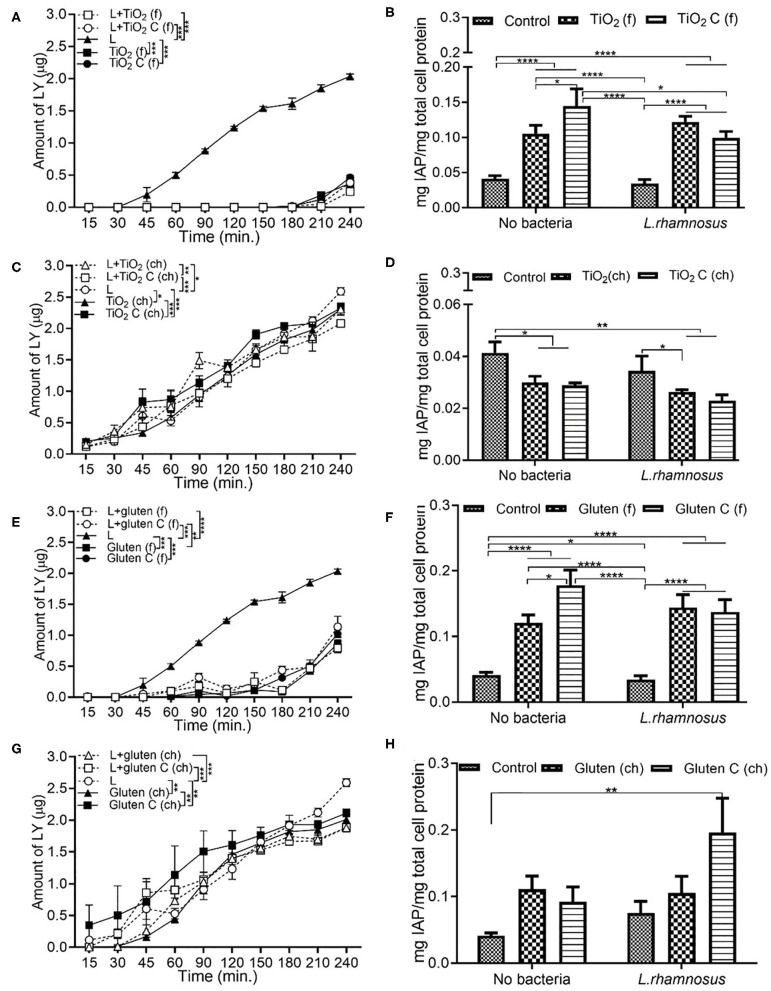
Additive source affects the IAP activity. The Caco-2/HT29-MTX intestinal model was exposed to media (DMEM subjected to *in vitro* digestion, serves as overall control) or food grade (f) or chemical grade (ch) additives and 10^3^ CFU/mL *L. rhamnosus* for 4 h. TiO_2_ (f) or TiO_2_ C (f) **(A)** Permeability **(B)** IAP activity. TiO_2_ (ch) and TiO_2_ control (ch) **(C)** Permeability **(D)** IAP activity. Gluten (f) and gluten control (f) **(E)** Permeability **(F)** IAP activity. Gluten (ch) and gluten control (ch). **(G)** Permeability **(H)** IAP activity. Error bars represent SEM. FDR-adjusted *p*-values: *P < 0.05; **P < 0.01; ***P < 0.001; ****P < 0.0001 using linear regression with FDR correction for permeability assay and two-way ANOVA with FDR correction for IAP assay. The data was collected from two experimental replicates with four (permeability assay) and 12 (IAP assay) biological replicates each. C, control; L, *L. rhamnosus*.

Similar to the permeability results observed in section Chemical Grade Additives Induce Higher Permeability Than Food Grade Additives, the introduction of food grade additives led to a lower permeability compared to *L. rhamnosus* alone. A significantly low permeability was observed with food grade TiO_2_, both in the presence and absence of *L. rhamnosus* (Figure 6A). In comparison to the DMEM control, the IAP activity increased upon exposure to food grade TiO_2_, followed by food grade TiO_2_ control, both with and without *L. rhamnosus* (Figure 6B). Even in when exposed with chemical grade additives, the presence of *L. rhamnosus* led to a higher permeability independently, which was seen to decrease in combination with chemical grade TiO_2_ (Figure 6C). As opposed to food grade TiO_2_, IAP activity in the presence of chemical grade TiO_2_ was reduced both in the presence and absence of *L. rhamnosus*, compared to the DMEM control (Figure 6D). Food grade gluten followed the same permeability and IAP activity trend as food grade TiO_2_. Presence of *L. rhamnosus* showed the highest permeability, which significantly reduced in combination with food grade gluten (Figure 6E). IAP activity was increased with addition of food grade gluten when compared to DMEM controls (Figure 6F). In chemical grade form, *L. rhamnosus* with gluten showed a significantly higher permeability than gluten alone (Figure 6G). In comparison to control (DMEM), the IAP activity increased with exposure to chemical grade gluten (Figure 6H). The high IAP activity in the presence of chemical grade gluten control and *L. rhamnosus* correlates to the low permeability observed under the same conditions.

### *E. coli* Produces Indole Under Low Permeability Conditions

**Figure 7:**
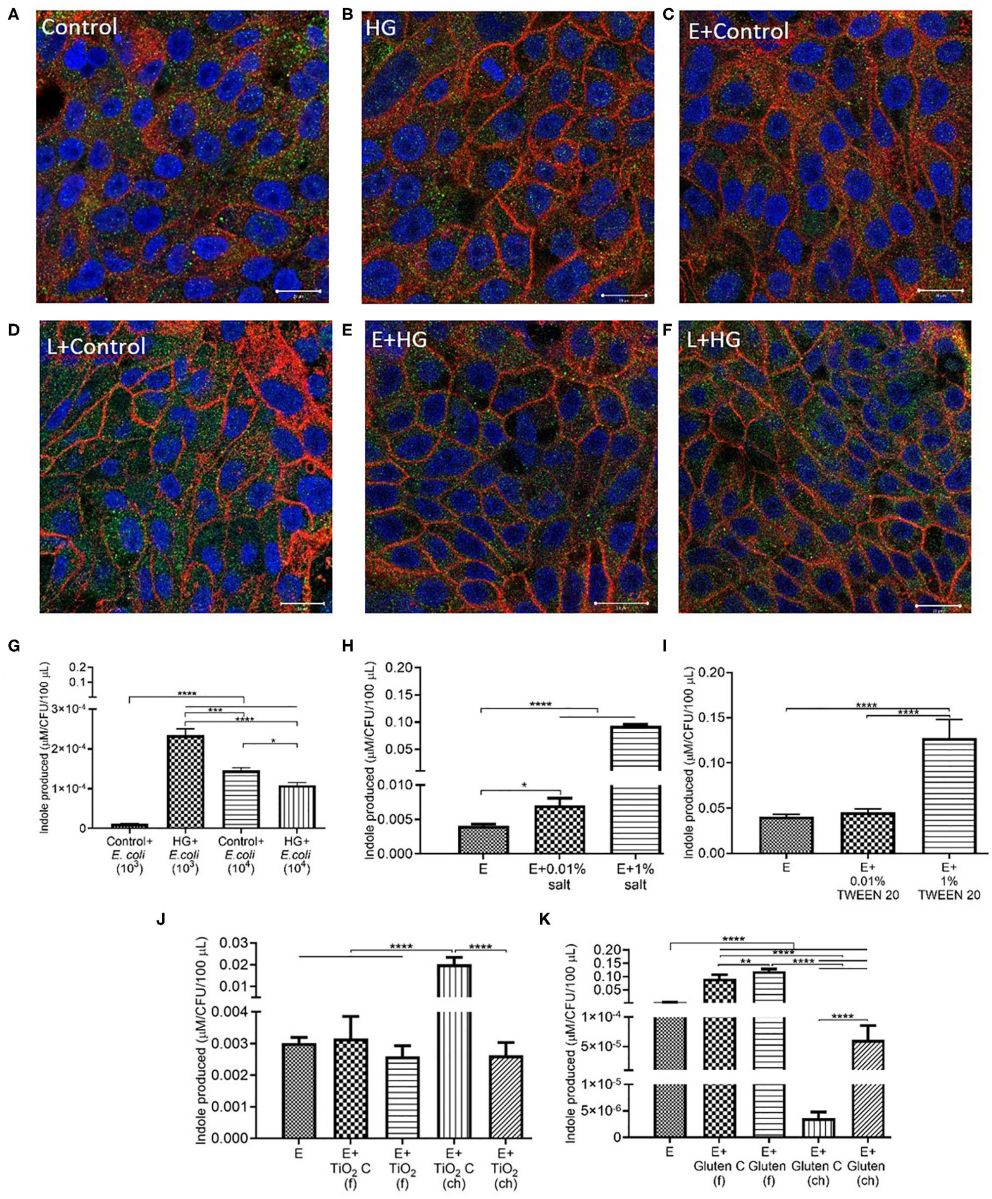
*E. coli* produces indole in low permeability conditions. Representative confocal images of the *in vitro* epithelium stained with immunofluorescence for occludin (red), ZO-1 (green), integral plasma membrane proteins located at the tight junctions, and DNA (blue) after 4 h exposure to **(A)** control (5 mM glucose + 20 mM mannitol) **(B)** High glucose (25 mM DMEM, HG) **(C)** control (5 mM glucose + 20 mM mannitol) and 10^3^ CFU/mL *E. coli* **(D)** control (5 mM glucose + 20 mM mannitol) and 10^3^ CFU/mL *L. rhamnosus* **(E)** HG (25 mM DMEM) and 10^3^ CFU/mL *E. coli* **(F)** HG (25 mM DMEM) and 10^3^ CFU/mL *L. rhamnosus*. Imaged at 63×-oil immersion. Scale bars are 20 μm. **(G,H)** Amount of indole produced by 10^3^ CFU/mL *E. coli* after a 4 h exposure to **(G)** control (5 mM glucose + 20 mM mannitol) and HG (25 mM DMEM) with 10^4^ CFU/mL *E. coli* **(H)** 0.01 and 1% salt **(I)** 0.01 and 1% TWEEN 20 **(J)** TiO_2_ (food, f), TiO_2_ control (f), TiO_2_ (chemical, ch) or TiO_2_ control (ch) **(K)** gluten (f), gluten control (f), gluten (ch), and gluten control (ch). Error bars represent SEM. FDR-adjusted *p*-values: *P < 0.05; **P < 0.01; ***P < 0.001; ****P < 0.0001 using one-way ANOVA with FDR correction for indole estimation. The data was collected from two experimental replicates with five (indole estimation) biological replicates each. C, control; E, *E. coli*; L, *L. rhamnosus*.

The Caco-2/HT29-MTX monolayer was immuno-stained to visualize the tight junction proteins occludin and ZO-1. Control (5 mM glucose + 20 mM mannitol) and high glucose (25 mM DMEM) conditions showed no change in tight junction protein distribution following exposure (Figures 7A,B). The addition of *E. coli* and *L. rhamnosus* to control and HG conditions did not contribute to a change in the distribution of tight junction proteins (Figures 7C–F). There was no significant change in the area covered by each TJ protein/total cells in the frame (Figure S4A).

A higher indole production was detected in inoculum of 10^3^ CFU/mL *E. coli* than 10^4^ CFU/mL *E. coli* under HG conditions (Figure 7G). In the case of salt (Figure 7H) and TWEEN 20 (Figure 7I), the highest inoculum concentration resulted in a higher indole production. Chemical grade TiO_2_ showed the highest indole production. *E. coli* produced the same level of indole in food grade and chemical grade TiO_2_ conditions (Figure 7J). Food grade gluten resulted in higher indole production compared to chemical grade gluten (Figure 7K).

### *L. rhamnosus* Alters the Tight Junction Protein Distribution

**Figure 8:**
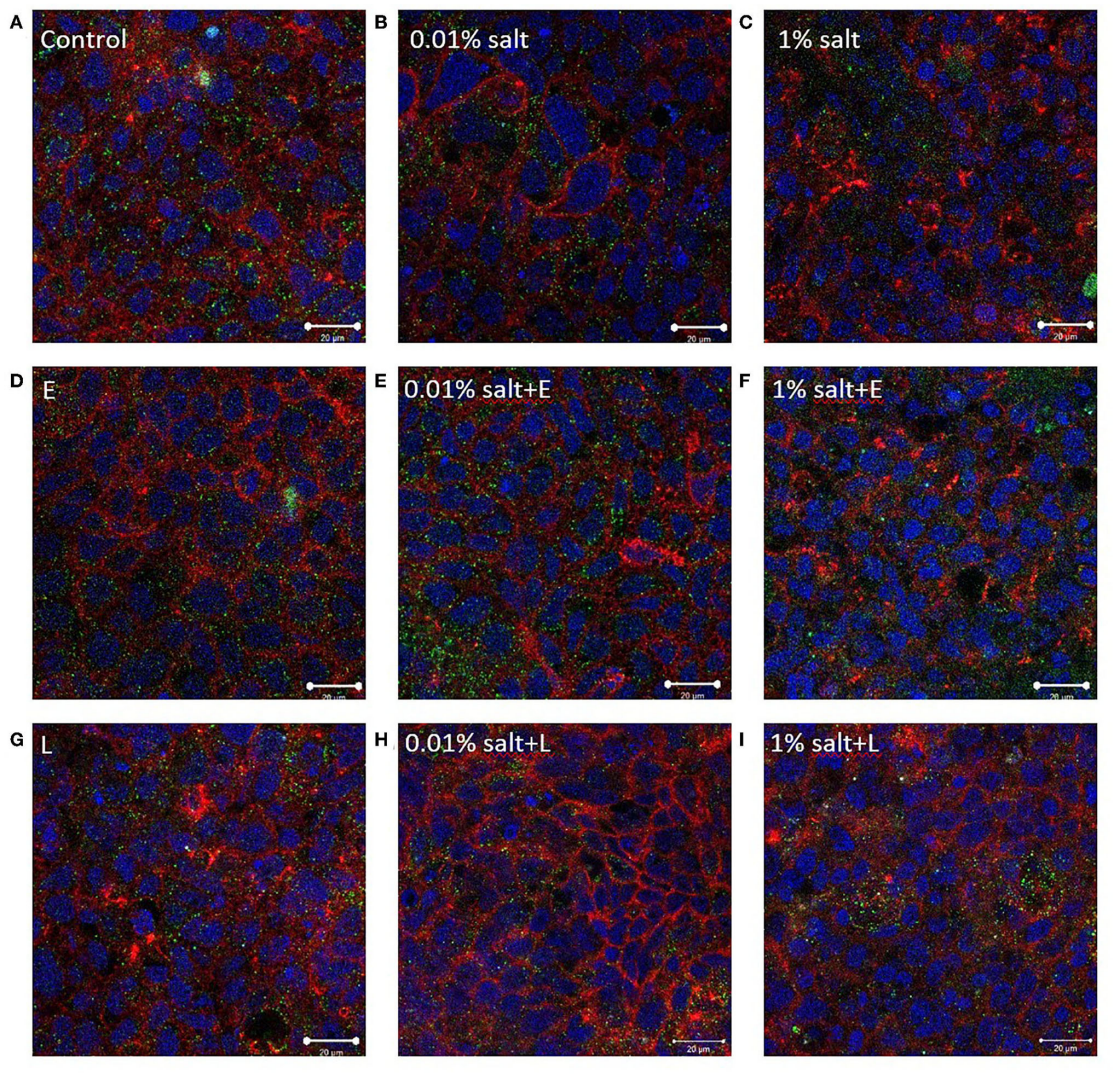
*L. rhamnosus* alters the tight junction protein distribution. Representative confocal images of the *in vitro* epithelium stained with immunofluorescence for occludin (red), ZO-1 (green), integral plasma membrane proteins located at the tight junctions, and DNA (blue) after 4 h exposure to **(A)** control (media) **(B)** 0.01% salt **(C)** 1% salt **(D–F)** with 10^3^ CFU/mL *E. coli* and **(G–I)** with 10^3^ CFU/mL *L. rhamnosus*. Imaged at 63×-oil immersion. Scale bars are 20 μm. E, *E. coli*; L, *L. rhamnosus*.

Compared to controls, immunocytochemistry did not show any prominent change in occludin or ZO-1 distribution in the presence of salt (Figures 8A–C). In combination with *E. coli*, the TJ distribution did not show any change (Figures 8D–F). However, an increase in occludin and ZO-1 was observed upon addition of *L. rhamnosus* to the salt conditions (Figures 8G–I), which was supported by the ImageJ analysis (Figure S4B).

## Discussion

This study explores the interactions between human dietary intake, intestinal function, and individual bacterial species in an *in vitro* model of the small intestine. Dietary habits have the ability to change the natural microbiota in the gut (24). A healthy human adult houses trillions of gut microbial cells. Under normal conditions these microorganisms assist in the maintenance of intestinal health, thus a change in the microbiota can jeopardize normal intestinal function. With a growing Westernization of diets around the world, increasing amounts of food additives and preservatives are being used and ingested by the human population. These additives are used to enhance the taste, aesthetics, and/or shelf life of the food products (25). With this increased consumption, it is essential to determine the effects of these added chemicals on human gut health and function. Previous studies using *in vitro* (15, 19, 26–28) and *in vivo* (15, 29–31) models have established that food additives directly influence the intestinal health and function of the lower GI tract. Different bacterial species are genetically more suitable to utilize specific substrates. Thus, dietary intake can potentially influence microbiota composition (32). A recent study conducted with human patients showed an inverse correlation between small intestinal permeability and microbial diversity (33). A change in dietary pattern (from high fiber to low fiber, high sugar diet) resulted in decrease in microbial diversity (33). However, there is a lack of *in vitro* studies that analyze the cellular scale effects of these food additive-derived changes in barrier function. An *in vitro* model provides a straightforward method to study these effects.

Intestinal alkaline phosphatase (IAP), a gut mucosal defense factor, is involved in the regulation of intestinal pH by production of bicarbonate ions. IAP production is induced during the establishment of the gut microbiota, and the presence of IAP is important for the maintenance of a healthy gut environment. IAP has also been documented to improve the microbiota population (34) and decrease expression of TNFα (8) in intestinal models. This was previously demonstrated where IAP knockout mice presented lower fecal bacterial population compared to wild type mice, particularly in regard to *Lactobacilli* and *E. coli* (34).

According to a study in 2003 by D'Souza et al., exposure of Caco-2 cell monolayers to 25 mM glucose led to a decrease in TEER and disruption of the F-actin ring (35). This study was the basis for selection of the glucose control conditions in our study. The normal human physiological level of fasting plasma blood glucose level is 5–5.5 mM/L, which matches the control conditions (5 mM glucose). Mannitol, structurally, and chemically similar to glucose, was added at 20 mM, to ensure that changes in the intestinal permeability were not a result of differences in osmolarity. The recommended blood glucose level of a diabetic patient falls in the range of 10–20 mM/L (36), therefore the *in vitro* 25 mM glucose presented a worst-case scenario. Here, we found that when using the *in vitro* monolayer, of the five food additives discussed in this paper, high glucose (HG) diet had the most detrimental effect on permeability and IAP (Figures 1A, 3A, 5A). Compared to control conditions, 0.65 μg more LY was transferred across the monolayer in HG condition. The increase in permeability due to HG was accompanied by a decrease in IAP activity as well (not significant), which was further reduced in combination with TNFα (Figure 1B, FDR-adjusted *p* < 0.01). TNFα has been reported to inhibit IAP gene expression by possibly blocking the histone acetylation in its promoter (34). While the combination of HG and TNFα does not show a significant negative effect on permeability, it poses a combined negative effect on the IAP activity.

Administration of controlled amounts of live microorganisms in the form of probiotics, has proven to be beneficial to the host health (37). *L. rhamnosus* GG is the most researched probiotic strain for its beneficial properties in regulation of gut health (38). The same strain was used in this study with the hypothesis that the negative effects of food additives will be reversed by the addition of *L. rhamnosus* to the *in vitro* system. Along with *L. rhamnosus, E. coli*, a Gram-negative bacterium was also used. *E. coli* is primarily present in the colon; however, the distal ileum has been shown to contain more Gram-negative than Gram-positive bacteria (5). Our previous work demonstrated increased intestinal permeability in the *in vitro* Caco-2/HT29-MTX model, and a reduced gut size in *Drosophila melanogaster* model in response to a high sugar diet, which was rectified with *L. rhamnosus* (15). Similarly, in this study we found that intestinal permeability was significantly reduced upon exposure to *L. rhamnosus* (Figure 5A) and *E. coli* (Figure 3A) under HG conditions.

Inflammatory bowel disease (IBD) and celiac disease patients show increased paracellular permeability and altered expression of ZO proteins, suggesting an association between TJ proteins and intestinal disease (39). Due to the significant role of TJ proteins in regulation of the dynamic nature of the TJ, changes in the expression or distribution of these proteins can disrupt the integrity of intestinal barrier. This may potentially allow passage of bacterial toxins from the lumen to the bloodstream, which can lead to both food allergies and inflammation. Transport between intestinal epithelial cells is regulated by the tight junction proteins such as junctional adhesion molecules (JAM), occludins, and claudins, connected to a network of zonula occludens (ZO-1, ZO-2, ZO-3). Out of the three ZO proteins, ZO-1 has been the most studies and is documented to facilitate TJ protein assembly. Previously it has been reported that occludin shows significant involvement in maintenance and assembly of the TJ, and it is the most reliable marker for immunohistochemical analysis of TJ (40). Knockdown of occludin causes an increase in permeability across the epithelial barrier (39). Similar results were obtained in our study where the immunostained images of the monolayer showed an increase in occludin under *L. rhamnosus* + HG condition (Figure 7F), reflecting the reduced permeability seen across the intestinal monolayer. This suggests that restoration of TJ protein could be the mode of action to reinstate gut health in this model. Under the glucose control conditions, *L. rhamnosus* had no significant effect on permeability, IAP activity, and tight junction protein distribution, which may provide a model of normal physiological conditions of the gut.

In addition to dietary changes, it is also important to understand how changes in microbial concentration in the gut affect nutrient absorption and/or gut function. In order to mimic this biological phenomenon, the *in vitro* system was in presence of a higher initial bacterial concentration of 10^4^ CFU/mL. Since HG showed the highest permeability increase compared to control, it was selected to represent “the worst-case scenario” for this set of experiments. While the presence of 10^4^ CFU/mL *E. coli* significantly reduced the intestinal permeability in the HG condition (Figure S2A), *L. rhamnosus* did not contribute any significant change in HG condition. An interesting change was seen in the glucose control condition, representing the physiological blood glucose level. Compared to the absence of bacteria control, 10^4^ CFU/mL *L. rhamnosus* + glucose control showed a higher permeability than 10^4^ CFU/mL *L. rhamnosus* + HG (Figure S2B). In all other experimental tests with glucose, HG was shown to have the most significant effect on permeability.

One of the mechanisms of gut health regulation by the microbiome is through the release of bacterial signals. One of such signals is indole, which is produced by *E. coli* through the breakdown of tryptophan. A study by Bansal et al. showed that upon exposure of human enterocyte HCT-8 cells to indole, the transepithelial resistance (TEER) of the cells increased (41). In addition, there was an upregulation of the expression of genes involved in epithelial barrier regulation, thus contributing to restoration of the epithelial barrier function (41). With the knowledge of the role that indole plays in regulation of TJ properties, the permeability trends in this study can be linked to the indole results. Even though the growth of *E. coli* at the end of 4 h exposure was higher in the HG + 10^4^ CFU/mL condition (Figure S3A), a higher decrease in permeability and increase in indole production was present with HG + 10^3^ CFU/mL *E. coli* (ΔLY = 0.83 μg) compared to HG + 10^4^ CFU/mL *E. coli* (ΔLY = 0.33 μg). Thus, inclusion of bacterial species to the *in vitro* model significantly contributes to the remediation of intestinal barrier integrity and therefore proves to be an integral part of the system. Since mammalian viability has been seen to reduce with increases in bacterial concentration, in all other work 10^3^ CFU/mL of bacterial inoculum were used.

Salt is a common food additive used in the food industry for thickening, preservation of food, and/or to enhance the taste. Emulsifiers are used in food such as bakery products, ice creams, sauces, and coffee to improve texture and stability. According to the US Food and Drug Administration (FDA), a person should consume 2,300 mg of sodium per day; however, the average daily intake of an American is 3,400 mg of sodium per day (42). Up to 3.5% salt content (43) and 4.5 % emulsifier (44) is present in processed foods by weight, which formed the basis of 1% of salt and 1% TWEEN 20 used in this study. The salt and TWEEN 20 solutions were prepared in DMEM. The lower concentrations of salt and TWEEN 20 represent the physiologically relevant concentration of intake of the additives. A recent study showed that a high salt diet causes an increase in expression of pro-inflammatory genes and suppression of many cytokine genes in a mouse model (29). Our Caco-2/ HT29-MTX model showed no change in permeability with 1% salt compared to the control condition (DMEM). The IAP activity was significantly increased with 1% salt, both in presence and absence of TNFα (Figure 1D). The same trend has also been documented in a past *in vivo* study where high salt conditions were seen to enhance the activity of IAP in the intestinal mucosa of rainbow trout (45). The IAP enzyme has been associated with the ability to adapt its activity in high salt conditions (45). However, contradictory results have been reported in a more recent study using a rat model. High salt concentration led to a decrease in IAP activity in duodenal samples from the rat (46). A high amount of salt can cause an osmotic pressure in the system, suppressing the bacterial growth. Even though *E. coli* is non-halophilic, it can survive and adapt in a high salt (up to 11%) environment (47). *E. coli* survival in presence of 1% salt was observed with a bacterial viability assay, which was accompanied by increased indole production by *E. coli* at higher salt concentration. This indicates that the negative effects of salt are potentially being regulated through indole production in this model. The indole results suggest a relation between the decrease in permeability and increased indole production by *E. coli*. The positive impact of indole on TJ resistance was published by Bansal et al. (41), outlining the association between E. coli produced indole and reduced inflammation and improved epithelial barrier. The same phenomenon could be occurring in our model, however, a mechanistic study would be required for confirmation. The increase in occludin and ZO-1 in the presence of 1% salt and *L. rhamnosus* (Figure 8I), indicates that restoration of TJ protein could be the mode of action of *L. rhamnosus* to maintain gut health. This was also supported by an increase in IAP activity with *L. rhamnosus* + 1% salt condition (Figure 5D). The continued growth of bacteria in presence of different food additives, along with permeability data showing that the changes observed in intestinal function are a result of association between bacteria and dietary additives and not overpopulation or death of bacteria. Pro-inflammatory cytokines such as TNFα can inhibit the expression of the *ALPI* gene (responsible for coding of IAP) in HT29 cells (34). This model shows that under stressful or inflamed conditions, increasing TWEEN 20 concentration reduces the permeability and IAP activity. The same trend was shown in presence of bacteria. Compared to the control condition (DMEM), bacteria + 1% TWEEN 20 resulted in the lowest permeability (Figures 3E, 5E). This was also reflected in the amount of indole produced by *E. coli*, which was higher with 1% TWEEN 20 (Figure 7I). A study published in 2018 showed a change in mucus thickness in an *in vivo* (mice) and *in vitro* model of the small intestine in response to emulsifiers. The emulsifiers also led to a change in the swimming speed of *E. coli* present in the model (48). Chassaing et al. observed that altered microbiota composition and reduced mucus thickness in mice subjected to TWEEN 80, led to translocation of bacteria closer to the epithelial barrier (49). According to the bacterial viability study, *E. coli* and *L. rhamnosus* growth was increased with increasing TWEEN 20 concentration (Figure S2C). The type of emulsifier used in the current study does not impact the barrier permeability, but enhanced the bacterial growth and reduced the IAP activity, showing that it can affect the microbiota population and influence intestinal regulatory mechanisms.

Food grade-TiO_2_ NPs, identified as E171, are widely used as whitening agents in hard shelled candies, chewing gums, and chocolates. In the US, an average adult consumes 0.2–0.7 mg TiO_2_/kg_bw_/day (16). As 36% of the E171 particles are in the nano-range, this results in a large exposure to nano-TiO_2_ (16)_._ Gluten, a major component of wheat, can trigger an inflammatory response in the small intestine of celiac disease patients (27). TiO_2_ NP and gluten have been documented to trigger an inflammatory response in the small intestine (19, 27). While TiO_2_-NPs have been shown to reduce intestinal barrier functions, increase pro-inflammatory signaling, and IAP activity (19), very few studies have used food-based NP. In our Caco-2/HT29-MTX model, food grade additives showed a significantly low permeability and IAP activity. Under non-inflammatory conditions, introduction of food grade TiO_2_ and gluten decreased the IAP activity compared to control (DMEM medium), but increased in combination with TNFα (Figures 2B,F). As opposed to all other food additives used in this study, TiO_2_ and gluten were digested *in vitro* prior to exposure study. There was no significant change in permeability with TNFα + food grade TiO_2_ and gluten condition compared to the no TNFα condition (Figures 2A,C), but the addition of food grade additive led to a significant decrease in permeability when compared to the control. The design of the current study mimics the physiological scenario of dosage and mode of consumption of the nanoparticles and shows that an acute exposure to nanoparticles or gluten in their food grade form and after digestion does not impact the barrier integrity negatively. Upon addition of *E. coli*, neither food grade TiO_2_ nor gluten showed a significant change in IAP activity compared to the no bacteria conditions. Although gluten-free bread and TiO_2_ free M&M candies were used as controls for food-grade additives, the additional components in the samples could be contributing to the changes seen in intestinal permeability and IAP activity. These components may include different protein content of the two types of bread, additional sugar, fats, or other ingredients in the candies. Further analysis could be improved by using a pure, non-gluten prolamin protein source as a negative control for gluten. Chemical grade additives showed a different trend than their food grade counterparts. The overall amount of LY measured at the end of the 4 h exposure to chemical grade additives was double the amount seen with food grade additives. Independently, *E. coli* caused a higher permeability and IAP activity, which reduced in combination with chemical grade food additives.

Previous *in vivo* studies showed that gliadin, the component of gluten associated with intestinal damage, increases the intestinal permeability, and activates zonulin release (50, 51). The *in vivo* models used a higher dose of gluten, which would result in a more significant effect on the animal models. Our study showed low permeability and high IAP activity in food grade gluten conditions compared to no additive condition, indicating better gut function. The source of gluten impacted the total IAP activity observed. While the trend with food and chemical grade was the same, a lower IAP activity was observed with chemical grade gluten. This can be associated with a higher permeability seen with chemical grade gluten as compared to food grade gluten. Some bacterial species from the genera *Lactobacillus* and *Bifidobacterium* tend to be lower in abundance in celiac disease patients than in healthy individuals (37). These probiotic bacteria are known for their protective anti-inflammatory mechanisms and health benefits. *L. rhamnosus* has been documented to display proteolytic activity and perform as a dominant gluten-degrading bacteria (52). It has also been shown to regulate intestinal permeability and microbial dysbiosis in an experiment mouse model of sepsis (53). The low permeability in the model in the presence of bacteria and chemical grade TiO_2_ or gluten ascertains the beneficial effects of bacteria in this study. The highly viscous consistency of the food grade digests could potentially be a contributing factor in the low amount of LY passing across the barrier. The growth of *E. coli* was shown to increase in the presence of TWEEN 20 and chemical grade gluten when compared with other additives, while growth of *L. rhamnosus* increased in presence of salt, TWEEN 20, and food grade gluten. This shows that the bacterial growth can vary with dietary changes, suggesting an influence of diet on the microbial composition in the gut.

## Conclusion

The growing consumption of food additives may be associated with the increased occurrence of intestinal disorders. In this study an *in vitro* model was developed to test the interactions between small intestinal function, the upper GI microbiota, and food additive consumption. While HG showed a highly negative effect on intestinal permeability, the introduction of bacteria remediated those effects. Pristine NP or pure chemical grade gluten provides an indication of the effect of the additives, however, exposure of the model to these additives within a food matrix alters the final effects. The use of food grade products in this study provides a closer estimation on the effects of the additives in a more physiologically relevant context. The mode of action of the two type of bacteria tested in this model on intestinal health and function differed. While *L. rhamnosus* generally altered IAP or influenced TJ distribution, *E. coli* produced indole to strengthen the TJ. These roles may change due to differences in interaction between the bacterial species and the additives. Increased IAP activity in conditions with higher permeability indicates an attempt of the intestinal system to reinstate the homeostasis. The findings of this study emphasize the fact that different food substrates result in varied bacterial behavior, which can change the effect of the food substrates on the intestinal barrier. The GI model with bacteria and food additives within a food matrix is a step forward in development of a more realistic *in vitro* intestinal model with one of the major intestinal components, the microbiome.

## Data Availability Statement

The raw data supporting the conclusions of this article will be made available by the authors, without undue reservation.

## Author Contributions

MM, CM, and GM: conceptualization, methodology, data acquisition, data analysis, writing-review, and editing. GM, CM, and SS: funding acquisition. MM and SS: statistical testing. MM: writing-original draft. CM and GM: project administration. All authors contributed to the article and approved the submitted version.

## Conflict of Interest

The authors declare that the research was conducted in the absence of any commercial or financial relationships that could be construed as a potential conflict of interest.

## Supplementary Material

Figure S1 Permeability assay with 0.01% salt condition. The permeability of the Caco-2/HT29-MTX intestinal model was assessed following a 4 h exposure to control (DMEM) or 0.01% salt in combination with **(A)** 10 ng/mL TNFα (TNF) **(B)** 10^3^ CFU/mL *E. coli* or **(C)** 10^3^ CFU/mL *L. rhamnosus*. Error bars represent SEM. FDR-adjusted *p*-values: ^*^*P* < 0.05 using linear regression with FDR correction. The data was collected from two experimental replicates with four biological replicates each. TNF, TNF α; E, *E. Coli*; L, *L. rhamnosus*.

Figure S2 Permeability increased with higher *L. rhamnosus* concentration and HG. The Caco-2/HT29-MTX intestinal model was adapted to control (5 mM glucose + 20 mM mannitol) or high glucose (25 mM DMEM, HG) culture medium. **(A,B)** Lucifer yellow permeability was assessed in presence of **(A)** 10^4^ CFU/well *E. coli* **(B)** *L. rhamnosus* for 4 h. **(C,D)** IAP activity in presence of **(C)** 10^4^ CFU/well *E. coli* **(D)** *L. rhamnosus* for 4 h Error bars represent SEM. FDR-adjusted *p*-values: ^*^*P* < 0.05; ^**^*P* < 0.01 using linear regression with FDR correction for permeability assay and two way ANOVA with FDR correction for IAP assay. The data was collected from two experimental replicates with four (permeability assay) and 12 (IAP assay) biological replicates each. E, *E. coli*; L, *L. rhamnosus*.

Figure S3 Additives influence bacterial growth. Bacterial viability was quantified for the various culture conditions for 10^3^ CFU/mL inoculum of *E. coli* and *L. rhamnosus* and **(A)** Control (5 mM glucose + 20 mM mannitol) and high glucose (25 mM DMEM), along with 10^4^ CFU/mL inoculum of *E. coli* and *L. rhamnosus* **(B)** Control (medium), 0.01 and 1% salt **(C)** Control (DMEM), 0.01% TWEEN 20 and 1% TWEEN 20 **(D)** Control (DMEM), TiO_2_ (food, f), TiO_2_ C (f), TiO_2_ (chemical, ch) and TiO_2_ C (ch) **(E)** Control (DMEM), gluten (f), gluten C (f), gluten (ch) and gluten C (ch). Error bars represent SEM. FDR-adjusted *p*-values: ^*^*P* < 0.05; ^**^*P* < 0.01; ^***^*P* < 0.001; ^****^*P* < 0.0001 using two way ANOVA with FDR correction. The data was collected from two experimental replicates with three biological replicates each. Tw, TWEEN 20; HG, High glucose; C, control; E, *E. coli*; L, *L. rhamnosus*.

Figure S4 *L. rhamnosus* increases TJ protein distribution. Immunocytochemistry images were analyzed using ImageJ based on surface area covered by tight junction proteins occludin and ZO-1/total number of cells in the frame after a 4 h exposure of the Caco-2/HT29 MTX cell model to **(A)** Control (5 mM glucose + 20 mM mannitol) and high glucose (25 mM DMEM) in combination with 10^3^ CFU/mL *E. coli* or *L. rhamnosus*. **(B)** Control (DMEM), 0.01 and 1% salt in combination with 10^3^ CFU/mL *E. coli* or *L. rhamnosus*. ^*^FDR-adjusted *P*-values: ^*^*P* < 0.05; ^***^*P* < 0.001 using two-way ANOVA followed by FDR correction. HG, High glucose; E, *E. coli*; L, *L. rhamnosus*.
