# Supplementary figures and images for: Bacteria Remediate the Effects of Food Additives on Intestinal Function in an in vitro Model of the Gastrointestinal Tract
Source: Front Nutr. 2020 Aug 12;7:131. doi: 10.3389/fnut.2020.00131 (PMC7434930; doi:10.3389/fnut.2020.00131)

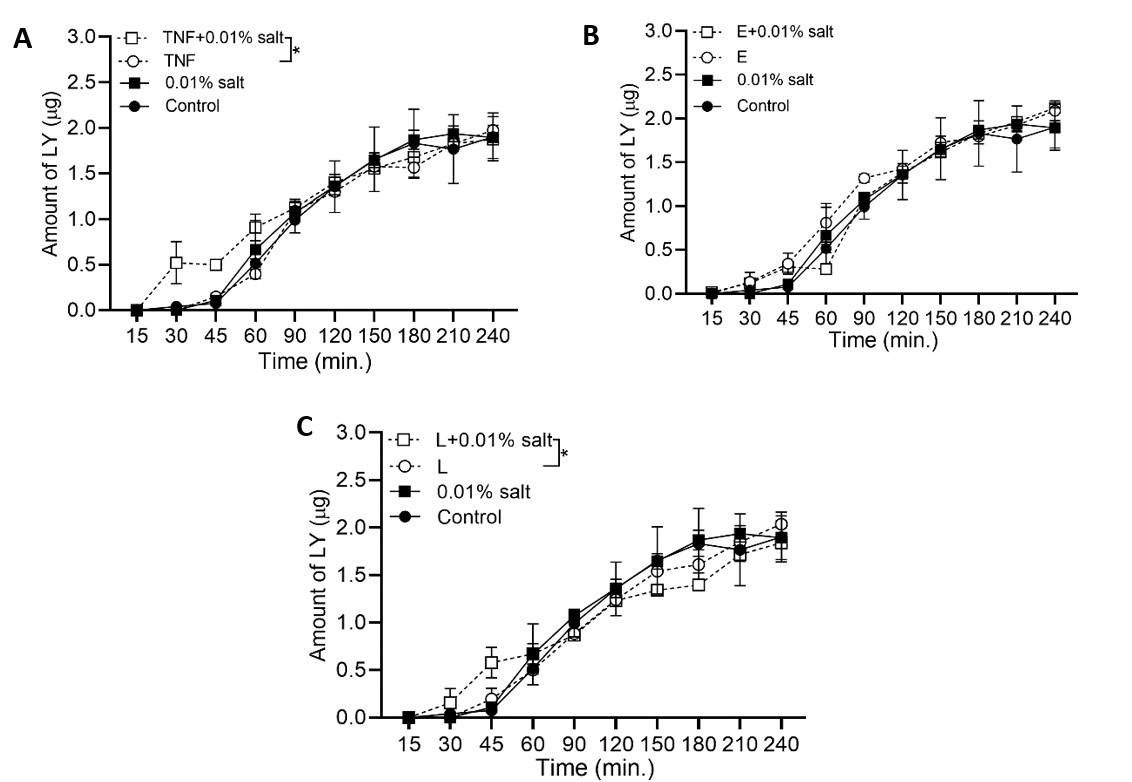

Supplement: Figure S1 — Permeability assay with 0.01% salt condition. The permeability of the Caco-2/HT29-MTX intestinal model was assessed following a 4 h exposure to control (DMEM) or 0.01% salt in combination with (A) 10 ng/mL TNFα (TNF) (B) 103 CFU/mL E. coli or (C) 103 CFU/mL L. rhamnosus. Error bars represent SEM. FDR-adjusted p-values: *P < 0.05 using linear regression with FDR correction. The data was collected from two experimental replicates with four biological replicates each. TNF, TNF α; E, E. Coli; L, L. rhamnosus. [file Image_1.JPEG]

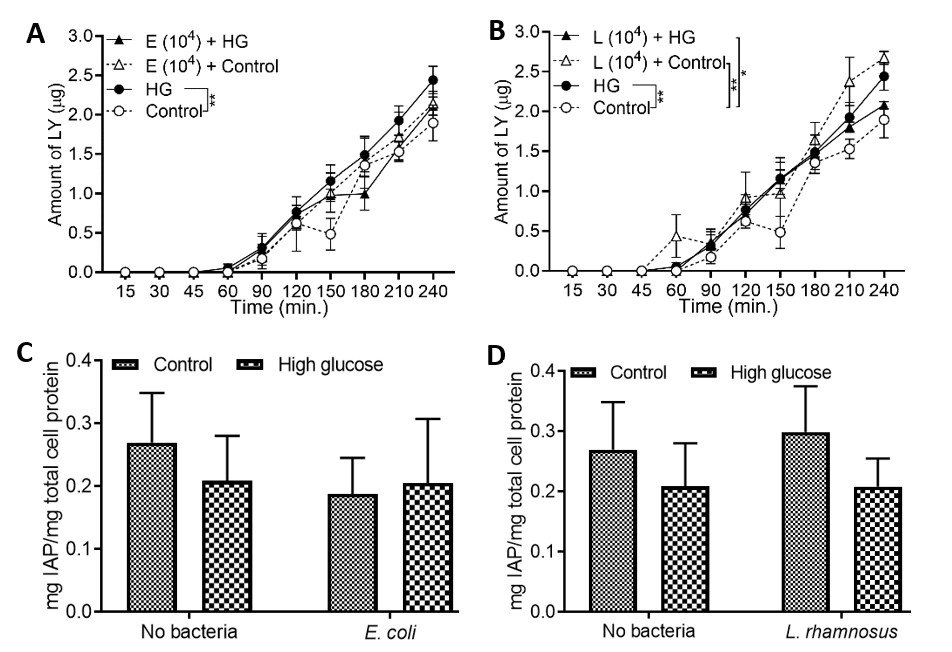

Supplement: Figure S2 — Permeability increased with higher L. rhamnosus concentration and HG. The Caco-2/HT29-MTX intestinal model was adapted to control (5 mM glucose + 20 mM mannitol) or high glucose (25 mM DMEM, HG) culture medium. (A,B) Lucifer yellow permeability was assessed in presence of (A) 104 CFU/well E. coli (B) L. rhamnosus for 4 h. (C,D) IAP activity in presence of (C) 104 CFU/well E. coli (D) L. rhamnosus for 4 h Error bars represent SEM. FDR-adjusted p-values: *P < 0.05; **P < 0.01 using linear regression with FDR correction for permeability assay and two way ANOVA with FDR correction for IAP assay. The data was collected from two experimental replicates with four (permeability assay) and 12 (IAP assay) biological replicates each. E, E. coli; L, L. rhamnosus. [file Image_2.JPEG]

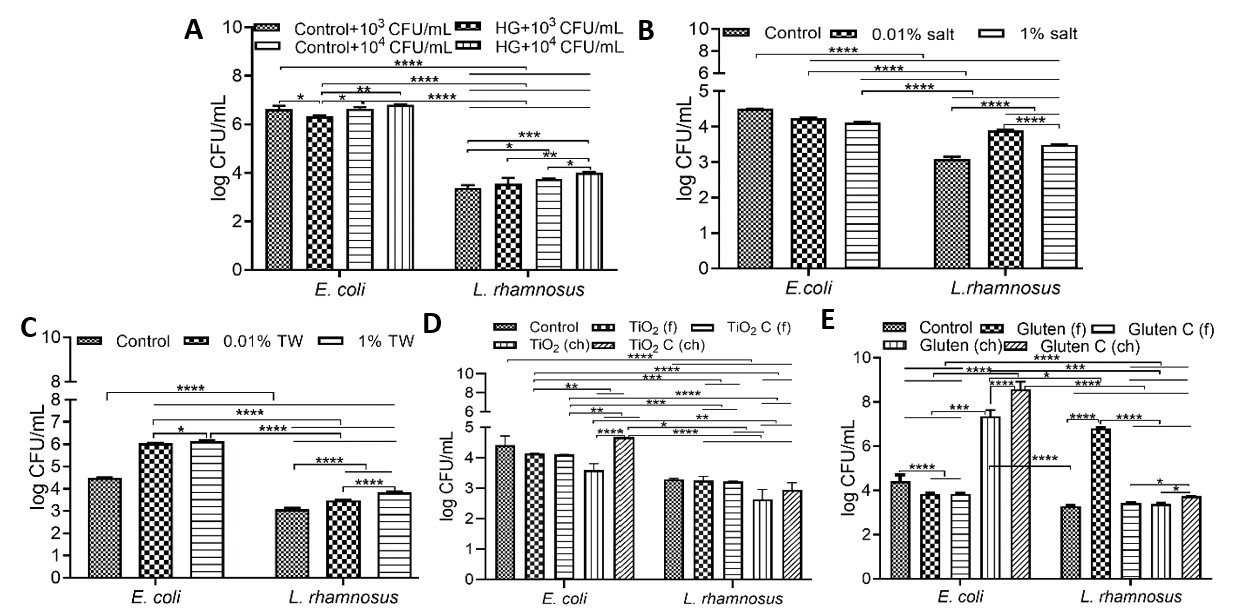

Supplement: Figure S3 — Additives influence bacterial growth. Bacterial viability was quantified for the various culture conditions for 103 CFU/mL inoculum of E. coli and L. rhamnosus and (A) Control (5 mM glucose + 20 mM mannitol) and high glucose (25 mM DMEM), along with 104 CFU/mL inoculum of E. coli and L. rhamnosus (B) Control (medium), 0.01 and 1% salt (C) Control (DMEM), 0.01% TWEEN 20 and 1% TWEEN 20 (D) Control (DMEM), TiO2 (food, f), TiO2 C (f), TiO2 (chemical, ch) and TiO2 C (ch) (E) Control (DMEM), gluten (f), gluten C (f), gluten (ch) and gluten C (ch). Error bars represent SEM. FDR-adjusted p-values: *P < 0.05; **P < 0.01; ***P < 0.001; ****P < 0.0001 using two way ANOVA with FDR correction. The data was collected from two experimental replicates with three biological replicates each. Tw, TWEEN 20; HG, High glucose; C, control; E, E. coli; L, L. rhamnosus. [file Image_3.JPEG]

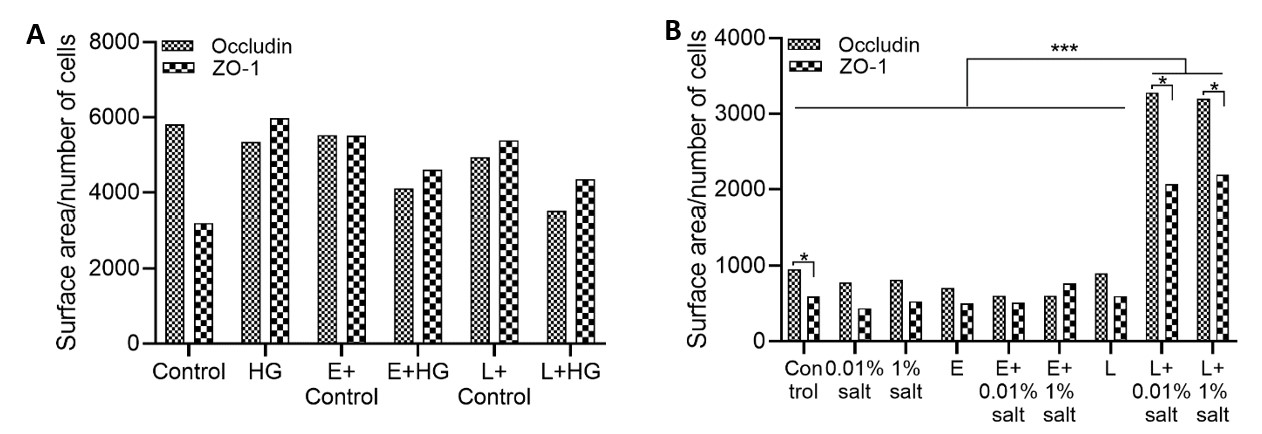

Supplement: Figure S4 — L. rhamnosus increases TJ protein distribution. Immunocytochemistry images were analyzed using ImageJ based on surface area covered by tight junction proteins occludin and ZO-1/total number of cells in the frame after a 4 h exposure of the Caco-2/HT29 MTX cell model to (A) Control (5 mM glucose + 20 mM mannitol) and high glucose (25 mM DMEM) in combination with 103 CFU/mL E. coli or L. rhamnosus. (B) Control (DMEM), 0.01 and 1% salt in combination with 103 CFU/mL E. coli or L. rhamnosus. *FDR-adjusted P-values: *P < 0.05; ***P < 0.001 using two-way ANOVA followed by FDR correction. HG, High glucose; E, E. coli; L, L. rhamnosus. [file Image_4.JPEG]
